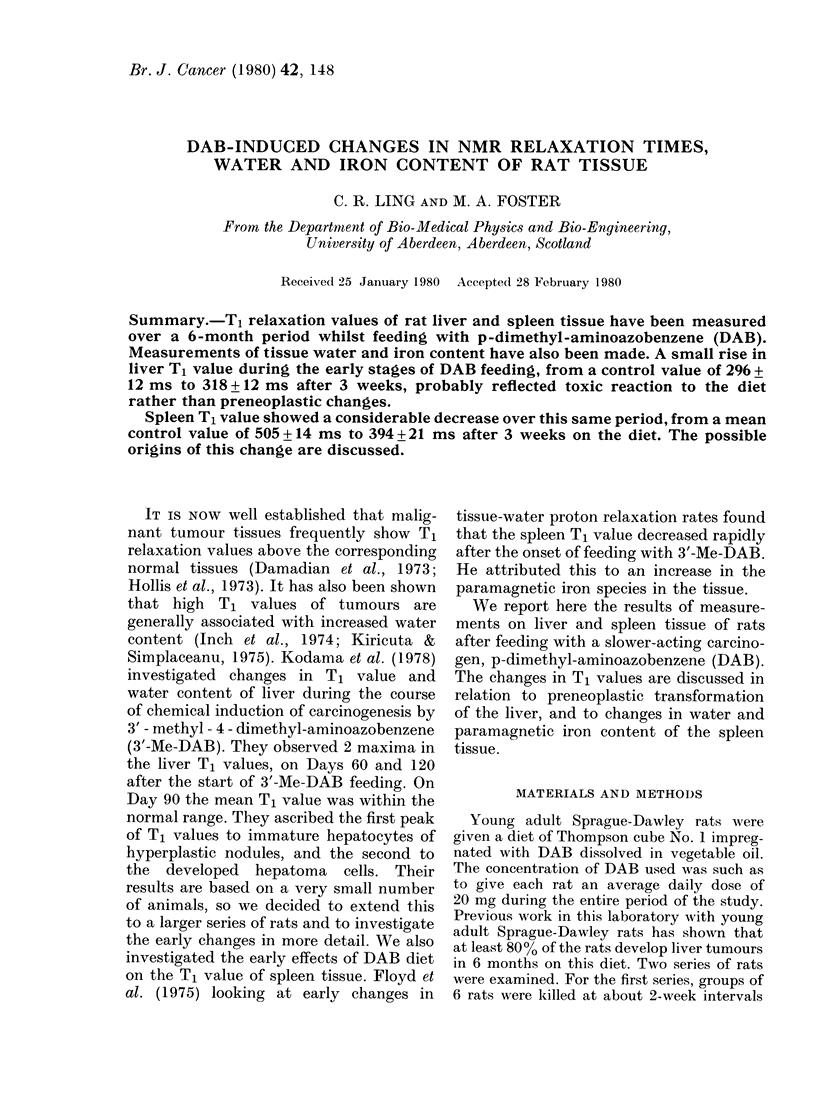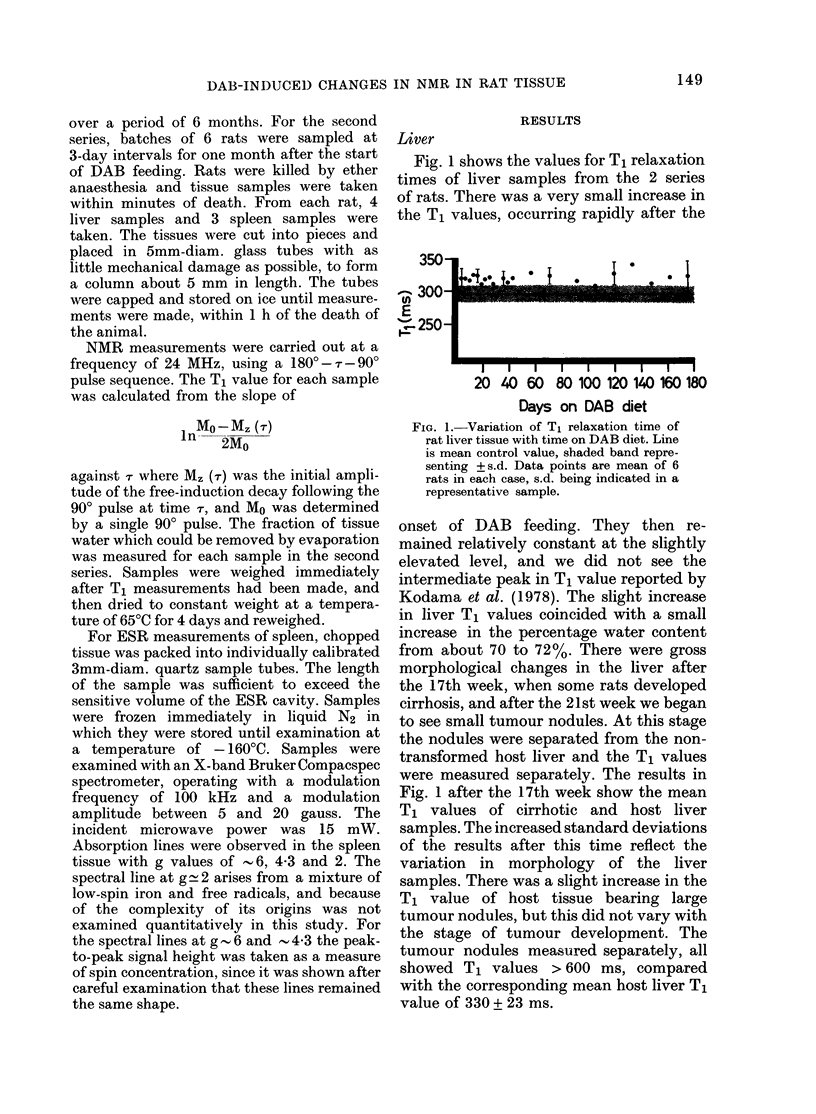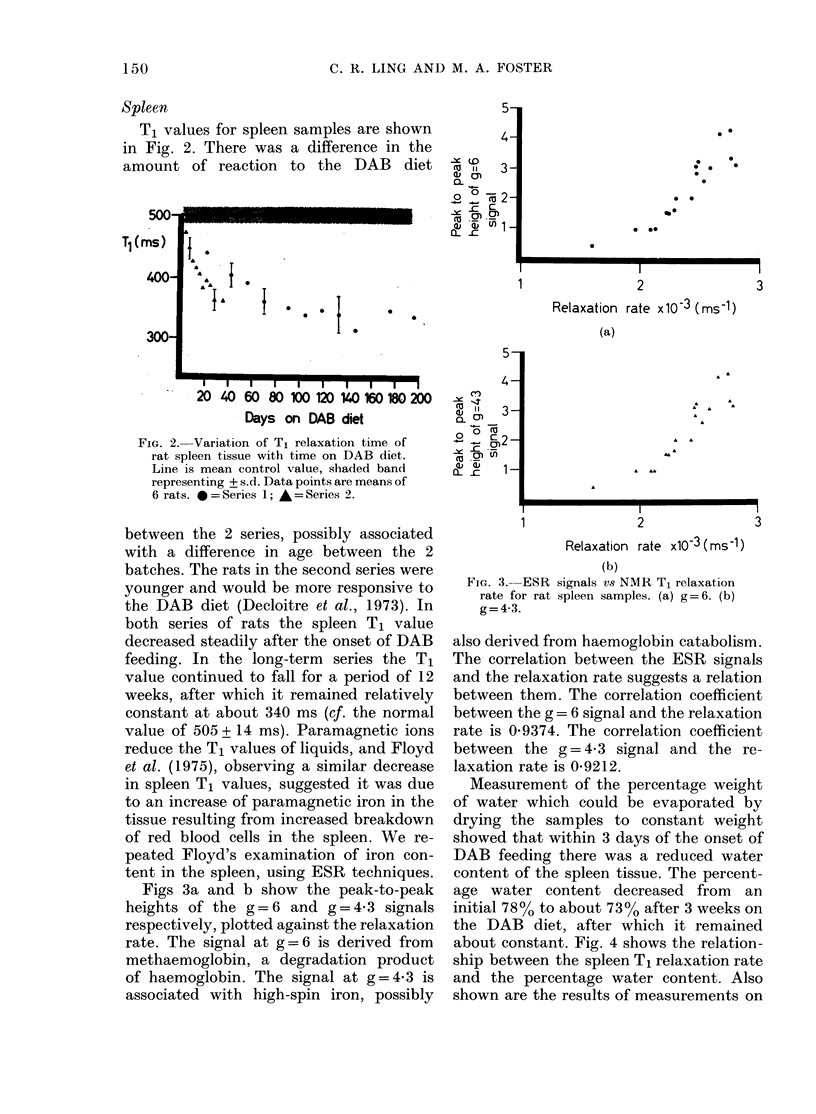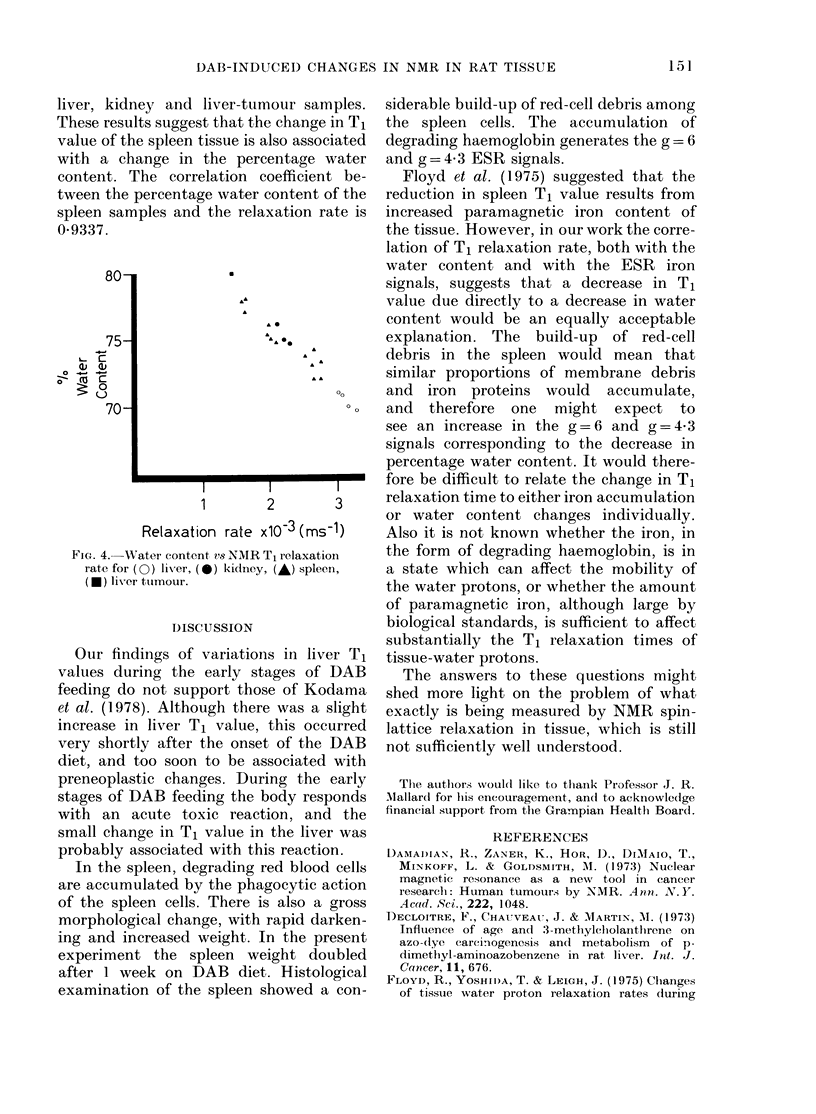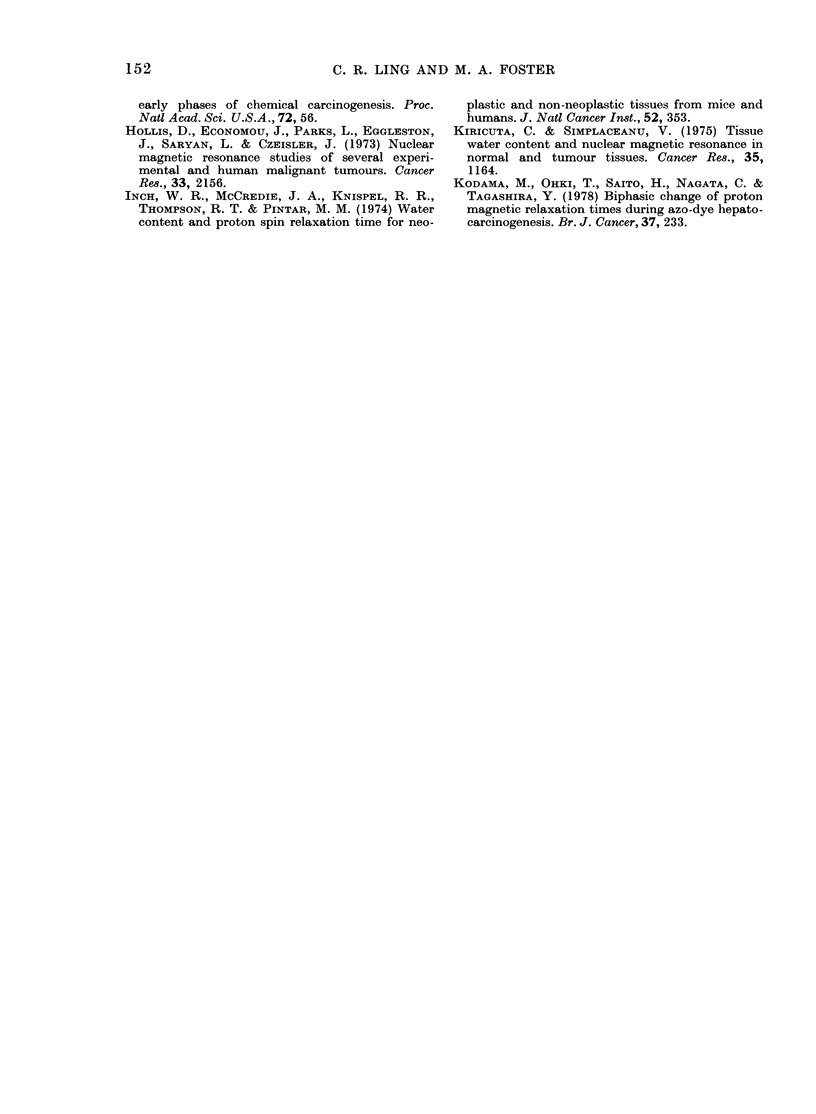# DAB-induced changes in NMR relaxation times, water and iron content of rat tissue.

**DOI:** 10.1038/bjc.1980.212

**Published:** 1980-07

**Authors:** C. R. Ling, M. A. Foster

## Abstract

T1 relaxation values of rat liver and spleen tissue have been measured over a 6-month period whilst feeding with p-dimethyl-aminoazobenzene (DAB). Measurements of tissue water and iron content have also been made. A small rise in liver T1 value during the early stages of DAB feeding, from a control value of 296 +/- 12 ms to 318 +/- 12 ms after 3 weeks, probably reflected toxic reaction to the diet rather than preneoplastic changes. Spleen T1 value showed a considerable decrease over this same period, from a mean control value of 505 +/- 14 ms to 394 +/- 21 ms after 3 weeks on the diet. The possible origins of this change are discussed.


*Br. J. Cancer* (1980) **42**, 148

### DAB-INDUCED CHANGES IN NMR RELAXATION TIMES, WATER AND IRON CONTENT OF RAT TISSUE

C. R. LING AND M. A. FOSTER

*From the Department of Bio-Medical Physics and Bio-Engineering, University of Aberdeen, Aberdeen, Scotland*

Received 25 January 1980 Accepted 28 February 1980

**Summary.—$T_1$ relaxation values of rat liver and spleen tissue have been measured over a 6-month period whilst feeding with p-dimethyl-aminoazobenzene (DAB). Measurements of tissue water and iron content have also been made. A small rise in liver $T_1$ value during the early stages of DAB feeding, from a control value of $296 \pm 12$ ms to $318 \pm 12$ ms after 3 weeks, probably reflected toxic reaction to the diet rather than preneoplastic changes.**

**Spleen $T_1$ value showed a considerable decrease over this same period, from a mean control value of $505 \pm 14$ ms to $394 \pm 21$ ms after 3 weeks on the diet. The possible origins of this change are discussed.**

IT IS NOW well established that malignant tumour tissues frequently show $T_1$ relaxation values above the corresponding normal tissues (Damadian *et al.*, 1973; Hollis *et al.*, 1973). It has also been shown that high $T_1$ values of tumours are generally associated with increased water content (Inch *et al.*, 1974; Kiricuta & Simplaceanu, 1975). Kodama *et al.* (1978) investigated changes in $T_1$ value and water content of liver during the course of chemical induction of carcinogenesis by 3′-methyl-4-dimethyl-aminoazobenzene (3′-Me-DAB). They observed 2 maxima in the liver $T_1$ values, on Days 60 and 120 after the start of 3′-Me-DAB feeding. On Day 90 the mean $T_1$ value was within the normal range. They ascribed the first peak of $T_1$ values to immature hepatocytes of hyperplastic nodules, and the second to the developed hepatoma cells. Their results are based on a very small number of animals, so we decided to extend this to a larger series of rats and to investigate the early changes in more detail. We also investigated the early effects of DAB diet on the $T_1$ value of spleen tissue. Floyd *et al.* (1975) looking at early changes in tissue-water proton relaxation rates found that the spleen $T_1$ value decreased rapidly after the onset of feeding with 3′-Me-DAB. He attributed this to an increase in the paramagnetic iron species in the tissue.

We report here the results of measurements on liver and spleen tissue of rats after feeding with a slower-acting carcinogen, p-dimethyl-aminoazobenzene (DAB). The changes in $T_1$ values are discussed in relation to preneoplastic transformation of the liver, and to changes in water and paramagnetic iron content of the spleen tissue.

### MATERIALS AND METHODS

Young adult Sprague-Dawley rats were given a diet of Thompson cube No. 1 impregnated with DAB dissolved in vegetable oil. The concentration of DAB used was such as to give each rat an average daily dose of 20 mg during the entire period of the study. Previous work in this laboratory with young adult Sprague-Dawley rats has shown that at least 80% of the rats develop liver tumours in 6 months on this diet. Two series of rats were examined. For the first series, groups of 6 rats were killed at about 2-week intervals over a period of 6 months. For the second series, batches of 6 rats were sampled at 3-day intervals for one month after the start of DAB feeding. Rats were killed by ether anaesthesia and tissue samples were taken within minutes of death. From each rat, 4 liver samples and 3 spleen samples were taken. The tissues were cut into pieces and placed in 5mm-diam. glass tubes with as little mechanical damage as possible, to form a column about 5 mm in length. The tubes were capped and stored on ice until measurements were made, within 1 h of the death of the animal.

NMR measurements were carried out at a frequency of 24 MHz, using a $180° - \tau - 90°$ pulse sequence. The $T_1$ value for each sample was calculated from the slope of

$$\ln\frac{M_0 - M_z(\tau)}{2M_0}$$

against $\tau$ where $M_z(\tau)$ was the initial amplitude of the free-induction decay following the 90° pulse at time $\tau$, and $M_0$ was determined by a single 90° pulse. The fraction of tissue water which could be removed by evaporation was measured for each sample in the second series. Samples were weighed immediately after $T_1$ measurements had been made, and then dried to constant weight at a temperature of 65°C for 4 days and reweighed.

For ESR measurements of spleen, chopped tissue was packed into individually calibrated 3mm-diam. quartz sample tubes. The length of the sample was sufficient to exceed the sensitive volume of the ESR cavity. Samples were frozen immediately in liquid $N_2$ in which they were stored until examination at a temperature of −160°C. Samples were examined with an X-band Bruker Compacspec spectrometer, operating with a modulation frequency of 100 kHz and a modulation amplitude between 5 and 20 gauss. The incident microwave power was 15 mW. Absorption lines were observed in the spleen tissue with g values of $\sim$6, 4·3 and 2. The spectral line at $g \simeq 2$ arises from a mixture of low-spin iron and free radicals, and because of the complexity of its origins was not examined quantitatively in this study. For the spectral lines at $g \sim 6$ and $\sim$4·3 the peak-to-peak signal height was taken as a measure of spin concentration, since it was shown after careful examination that these lines remained the same shape.

### RESULTS

### *Liver*

Fig. 1 shows the values for $T_1$ relaxation times of liver samples from the 2 series of rats. There was a very small increase in the $T_1$ values, occurring rapidly after the

FIG. 1.—Variation of $T_1$ relaxation time of rat liver tissue with time on DAB diet. Line is mean control value, shaded band representing ±s.d. Data points are mean of 6 rats in each case, s.d. being indicated in a representative sample.

onset of DAB feeding. They then remained relatively constant at the slightly elevated level, and we did not see the intermediate peak in $T_1$ value reported by Kodama *et al.* (1978). The slight increase in liver $T_1$ values coincided with a small increase in the percentage water content from about 70 to 72%. There were gross morphological changes in the liver after the 17th week, when some rats developed cirrhosis, and after the 21st week we began to see small tumour nodules. At this stage the nodules were separated from the non-transformed host liver and the $T_1$ values were measured separately. The results in Fig. 1 after the 17th week show the mean $T_1$ values of cirrhotic and host liver samples. The increased standard deviations of the results after this time reflect the variation in morphology of the liver samples. There was a slight increase in the $T_1$ value of host tissue bearing large tumour nodules, but this did not vary with the stage of tumour development. The tumour nodules measured separately, all showed $T_1$ values $> 600$ ms, compared with the corresponding mean host liver $T_1$ value of $330 \pm 23$ ms.

*Spleen*

$T_1$ values for spleen samples are shown in Fig. 2. There was a difference in the amount of reaction to the DAB diet

FIG. 2.—Variation of $T_1$ relaxation time of rat spleen tissue with time on DAB diet. Line is mean control value, shaded band representing ± s.d. Data points are means of 6 rats. ● = Series 1; ▲ = Series 2.

between the 2 series, possibly associated with a difference in age between the 2 batches. The rats in the second series were younger and would be more responsive to the DAB diet (Decloitre *et al.*, 1973). In both series of rats the spleen $T_1$ value decreased steadily after the onset of DAB feeding. In the long-term series the $T_1$ value continued to fall for a period of 12 weeks, after which it remained relatively constant at about 340 ms (*cf.* the normal value of 505 ± 14 ms). Paramagnetic ions reduce the $T_1$ values of liquids, and Floyd *et al.* (1975), observing a similar decrease in spleen $T_1$ values, suggested it was due to an increase of paramagnetic iron in the tissue resulting from increased breakdown of red blood cells in the spleen. We repeated Floyd's examination of iron content in the spleen, using ESR techniques.

Figs 3a and b show the peak-to-peak heights of the g = 6 and g = 4·3 signals respectively, plotted against the relaxation rate. The signal at g = 6 is derived from methaemoglobin, a degradation product of haemoglobin. The signal at g = 4·3 is associated with high-spin iron, possibly

FIG. 3.—ESR signals *vs* NMR $T_1$ relaxation rate for rat spleen samples. (a) g = 6. (b) g = 4·3.

also derived from haemoglobin catabolism. The correlation between the ESR signals and the relaxation rate suggests a relation between them. The correlation coefficient between the g = 6 signal and the relaxation rate is 0·9374. The correlation coefficient between the g = 4·3 signal and the relaxation rate is 0·9212.

Measurement of the percentage weight of water which could be evaporated by drying the samples to constant weight showed that within 3 days of the onset of DAB feeding there was a reduced water content of the spleen tissue. The percentage water content decreased from an initial 78% to about 73% after 3 weeks on the DAB diet, after which it remained about constant. Fig. 4 shows the relationship between the spleen $T_1$ relaxation rate and the percentage water content. Also shown are the results of measurements on liver, kidney and liver-tumour samples. These results suggest that the change in $T_1$ value of the spleen tissue is also associated with a change in the percentage water content. The correlation coefficient between the percentage water content of the spleen samples and the relaxation rate is 0·9337.

FIG. 4.—Water content *vs* NMR $T_1$ relaxation rate for (○) liver, (●) kidney, (▲) spleen, (■) liver tumour.

### DISCUSSION

Our findings of variations in liver $T_1$ values during the early stages of DAB feeding do not support those of Kodama *et al.* (1978). Although there was a slight increase in liver $T_1$ value, this occurred very shortly after the onset of the DAB diet, and too soon to be associated with preneoplastic changes. During the early stages of DAB feeding the body responds with an acute toxic reaction, and the small change in $T_1$ value in the liver was probably associated with this reaction.

In the spleen, degrading red blood cells are accumulated by the phagocytic action of the spleen cells. There is also a gross morphological change, with rapid darkening and increased weight. In the present experiment the spleen weight doubled after 1 week on DAB diet. Histological examination of the spleen showed a considerable build-up of red-cell debris among the spleen cells. The accumulation of degrading haemoglobin generates the $g = 6$ and $g = 4{\cdot}3$ ESR signals.

Floyd *et al.* (1975) suggested that the reduction in spleen $T_1$ value results from increased paramagnetic iron content of the tissue. However, in our work the correlation of $T_1$ relaxation rate, both with the water content and with the ESR iron signals, suggests that a decrease in $T_1$ value due directly to a decrease in water content would be an equally acceptable explanation. The build-up of red-cell debris in the spleen would mean that similar proportions of membrane debris and iron proteins would accumulate, and therefore one might expect to see an increase in the $g = 6$ and $g = 4{\cdot}3$ signals corresponding to the decrease in percentage water content. It would therefore be difficult to relate the change in $T_1$ relaxation time to either iron accumulation or water content changes individually. Also it is not known whether the iron, in the form of degrading haemoglobin, is in a state which can affect the mobility of the water protons, or whether the amount of paramagnetic iron, although large by biological standards, is sufficient to affect substantially the $T_1$ relaxation times of tissue-water protons.

The answers to these questions might shed more light on the problem of what exactly is being measured by NMR spin-lattice relaxation in tissue, which is still not sufficiently well understood.

The authors would like to thank Professor J. R. Mallard for his encouragement, and to acknowledge financial support from the Grampian Health Board.

### REFERENCES

DAMADIAN, R., ZANER, K., HOR, D., DIMAIO, T., MINKOFF, L. & GOLDSMITH, M. (1973) Nuclear magnetic resonance as a new tool in cancer research: Human tumours by NMR. *Ann. N.Y. Acad. Sci.*, **222**, 1048.

DECLOITRE, F., CHAUVEAU, J. & MARTIN, M. (1973) Influence of age and 3-methylcholanthrene on azo-dye carcinogenesis and metabolism of p-dimethyl-aminoazobenzene in rat liver. *Int. J. Cancer*, **11**, 676.

FLOYD, R., YOSHIDA, T. & LEIGH, J. (1975) Changes of tissue water proton relaxation rates during early phases of chemical carcinogenesis. *Proc. Natl Acad. Sci. U.S.A.*, **72**, 56.

HOLLIS, D., ECONOMOU, J., PARKS, L., EGGLESTON, J., SARYAN, L. & CZEISLER, J. (1973) Nuclear magnetic resonance studies of several experimental and human malignant tumours. *Cancer Res.*, **33**, 2156.

INCH, W. R., MCCREDIE, J. A., KNISPEL, R. R., THOMPSON, R. T. & PINTAR, M. M. (1974) Water content and proton spin relaxation time for neoplastic and non-neoplastic tissues from mice and humans. *J. Natl Cancer Inst.*, **52**, 353.

KIRICUTA, C. & SIMPLACEANU, V. (1975) Tissue water content and nuclear magnetic resonance in normal and tumour tissues. *Cancer Res.*, **35**, 1164.

KODAMA, M., OHKI, T., SAITO, H., NAGATA, C. & TAGASHIRA, Y. (1978) Biphasic change of proton magnetic relaxation times during azo-dye hepatocarcinogenesis. *Br. J. Cancer*, **37**, 233.